# Seroprevalence of Human Herpesvirus-8 in HIV-1 Infected and Uninfected Individuals in Cameroon

**DOI:** 10.3390/v5092253

**Published:** 2013-09-19

**Authors:** Christelle Mbondji-Wonje, Viswanath Ragupathy, Sherwin Lee, Owen Wood, Bih Awazi, Indira K. Hewlett

**Affiliations:** 1 Laboratory of Molecular Virology, Division of Emerging and Transmission Transmitted Diseases, Office of Blood Review and Research , Center for Biologics Evaluation and Research, Food and Drug Administration, 1401 Rockville Pike, Rockville, MD 20852, USA; 2 Chantal Biya International Reference Centre, Yaoundé, P.O Box 3077, Cameroon; 3 Laboratoire d’Analyses Médicales de Nkoldongo, Yaoundé P.O Box 20025, Cameroon

**Keywords:** HHV-8, HIV-1, serology, prevalence, Cameroon

## Abstract

We evaluated the prevalence of HHV-8 antibodies in 516 plasma samples collected from HIV positive and negative patients from blood banks and urban areas of Cameroon. Among HIV-1 positive samples, HHV-8 seropositivity rate was 61% based on combined reactivity using both ELISA and IFA techniques. HIV negative samples showed 62% seropositivity rate for HHV-8 antibodies. Our results indicate a high HHV-8 prevalence rate in both HIV infected and uninfected individuals in Cameroon.

## 1. Introduction

Human herpesvirus-8 (HHV-8) is an etiological agent of Kaposi Sarcoma (KS) [1]. It is also known as KSHV (Kaposi’s sarcoma-associated herpes virus). HHV-8 exists in KS tumor cells, certain rare lymphomas and multicentric Castleman’s disease [2]. The specific route of HHV-8 transmission remains in debate. However, previous reports indicate that HHV-8 could be transmitted through saliva, sexual contact, blood or blood products and organ transplant [3,4]. The prevalence of HHV-8 was higher in discrete geographic areas within Africa and the Eastern Mediterranean Basin [5]. These geographical locations reported the highest endemic KS prevalence prior to the HIV epidemic [6,7]. Endemic KS can occur as benign disease with few skin lesions or as an aggressive tumor involving lymph nodes. The fatal lymphadenopathic form of KS is common among young individuals in Africa [8]. Previous studies had demonstrated that cofactors such as HIV could increase the risk of development of HIV-KS and worsen its natural course [9,10]. In order to identify strategies to prevent and control HHV-8 related malignancies in HIV infected individuals, it is important to define the seroprevalence of HHV-8 in the African population. This pilot study focuses on seroprevalence of HHV-8/HIV-1 co-infection in Cameroon, a country in West Central Africa where the HIV epidemic has been well established [11] and divergent strains of HIV circulate [12,13]. The majority of HIV epidemiological studies conducted in Cameroon did not report prevalence of HHV-8 in the context of co-infection with HIV and general population studies on HHV-8 prevalence were shown to range from 38% to 62% [14,15,16]. None of these studies have reported prevalence rates in the setting of co-infection with HIV. In this preliminary report we present findings on the seroprevalence of HHV-8 among HIV infected and uninfected individuals. These results may provide insights into potential future public health impact of HHV-8 in the general population and in the context of HIV co-infection. 

## 2. Results and Discussion

### 2.1. Results

Out of the 516 samples analyzed in this study, 336 were HIV-1 positive and 180 were HIV negative. Of the 336 HIV-1 positive plasma, 236 (70%) were reactive with HHV-8 ELISA (Table 1), and 216 (64%) with HHV-8 IFA. Only 206 samples were concordantly positive with both ELISA and IFA, yielding a seroprevalence of 61% in HIV-1 positive patients. Of the HIV negative samples, 124 (69%) plasma specimens tested positive for HHV-8 antibodies by ELISA, while 129 (72%) samples yielded a positive result by IFA. Only 112 (62%) serum samples found to be dual positive in both assays (Table 1). No statistical differences were observed between seroprevalence rates of HHV-8 among HIV-1 infected and uninfected individuals (*p* > 0.05) (Table 1). 

**Table 1:** Seroprevalence of HHV-8 in HIV infected and uninfected individuals in Cameroon.

	HHV-8 Seropositive		
	HIV-1 infected individuals (N = 336)	HIV-1 uninfected individuals (N = 180)	*p* value Chi square
ELISA reactive (%)	236 (70%)	124 (69%)	0.7 (NS)
IFA reactive (%)	216 (64%)	129 (72%)	0.09 (NS)
Combined ELISA and IFA reactive (%)	206 (61%)	112 (62%)	0.9 (NS)

NS = non-significant

#### 2.1.1. Discussion

The present study is first to investigate the seroprevalence of HHV-8 in the Cameroonian population by considering their HIV-1 serostatus. In the present study, HHV-8 seroprevalence was found to be 61% and 62% among HIV-1 infected and uninfected individuals respectively, with no significant statistical difference between the two populations. HHV-8 seroprevalence in each group was as high as that found in several African populations independent of their HIV status. Indeed, seroprevalence rates higher than 60% have been reported in Uganda, Botswana and Tanzania [17]. Our findings are in accordance with the fact that, in Africa, the HIV epidemic has not had a significant impact on HHV-8 seroprevalence [17]. This result was not consistent with those reported in Nigeria and Ghana where the seroprevalence of HHV-8 was higher in HIV-1 infected adults and blood donors than in HIV negative individuals [18,19]. The observed differences in these studies could be due to differences in the performance characteristics of the lytic antigen ELISA assays (ABI, Columbia, MD, USA; Biotrin, Oxford, UK) used by both studies. Since there is no gold standard to determine HHV-8 serostatus, it has been demonstrated that seroprevalence rates among the general population may vary over a wide range depending on the diagnostic methods used [20]. Nonetheless, unlike previous studies, our study is limited by lack of demographic and clinical information, as our samples were a subset of anonymized samples collected for other purposes.

Since 1988, increased incidence of KS of about 3 to 20-fold has been reported in the Eastern and Southern part of Africa [17]. The use of HAART has considerably decreased the incidence of HIV-KS in developed countries [21]. However, in West and Central Africa, where HAART coverage is still low and new antiretroviral drugs are not available, KS remains one of the most frequent malignancies associated with HIV-1 [11,22]. In this regard, the high rate of HIV-1/HHV-8 that we observed indicates that HIV-KS could potentially become an increasing public health concern in Cameroon and warrants further investigation by future prospective studies in Cameroon and other countries in Africa. 

Despite repeated testing, we observed discrepancies between IFA and ELISA results. This may be because the tests measure sero-reactivity to different antigens that represent different stages of HHV-8 infection. In our study, by using either ELISA or IFA alone, the rate of anti-HHV-8 was higher than the estimated seroprevalence of 61%. It has been found that in contrast to lytic antigen assays, latent IFA was highly specific but not very sensitive [20]. Studies which intended to improve analytical performance of lytic ELISA and latent IFA by altering reaction parameters such as incubation times, sample dilution, or antigen concentration concluded that ELISA produced better detection of antibodies to the virus than the IFA [23]. Nevertheless, combining assays using lytic antigen with those using latent antigens may be a more appropriate approach for HHV-8 antibody detection in clinical samples as it may detect patients at multiple stages of infection [24,25]. Although PCR may be a more specific approach to detect HHV-8 infection, a large proportion of infected individuals do not have detectable HHV-8 DNA in peripheral blood [26]. We therefore chose to rely on assays targeting detection of antibodies. Further studies are needed to determine the suitability of current tests to reliably measure HHV-8 infection status and its prognostic value especially for HIV-1 infected individuals in whom KS remains one of the most prevalent neoplasms.

## 3. Experimental Section

### 3.1. Plasma Samples

We analyzed 516 archived plasma samples previously collected from 2002–2010 for other purposes [12,13]. Plasma was stored at −80 °C until analysis was performed. The initial HIV serostatus of these samples was based on the results of the Determine HIV1/2 (Abbott Laboratories, Diagnostics Division, Abbott Park, IL; USA), rapid assay in routine use in Cameroon. HIV serostatus was reconfirmed in our laboratory using FDA approved serologic assays according to the manufacturers’ instructions. For most of these samples we had limited demographic information. This study was approved through Institutional Review Board of the USFDA and has the exempt reference number 01-044B.

### 3.2. HHV-8 Assays

All samples were tested using both ELISA and the indirect IFA test for research use (Advanced Biotechnologies Inc., Columbia, MD, USA), according to manufacturer’s instructions. For ELISA (Cat No:15501000), the kit antigen was intended for the detection of IgG antibodies to lytic antigens of HHV-8. The IFA (Cat No:15330000) test detects antibodies to latent antigens of HHV-8. Each plasma sample was assayed in duplicate by ELISA. Discrepant samples were further retested in duplicate and repeatedly reactive samples were considered to be positive. Plasma samples were also tested by the IFA. A sample was considered as positive for HHV-8 if it was reactive using both assays. Seroprevalence estimation was based on combined ELISA and IFA positivity. 

### 3.3. Statistical Analysis

Data were analyzed using IBM SPSS version 19.0. Differences in variables were sought by chi square test, *p* < 0.05 was considered significant. 

## 4. Conclusions

In our study, antibodies to HHV-8 were detected at fairly high rates in both HIV-1 infected and uninfected individuals from Cameroon. Evaluation of HHV-8 seroprevalence is an important first step towards understanding the potential public health impact of HHV-8 infection in this population particularly among HIV-1 infected individuals. Future studies on HHV-8 monitoring in Cameroon may be needed to help improve our understanding of the pathogenesis of HIV-1/HHV-8 co-infection and to define strategies for prevention of HIV-KS.
